# Efficacy and Safety of Alogliptin in Patients With Type 2 Diabetes: Analysis of the ATTAK-J Study

**DOI:** 10.14740/jocmr2418w

**Published:** 2015-12-28

**Authors:** Hiroshi Takeda, Nobuo Sasai, Shogo Ito, Mitsuo Obana, Tetsuo Takuma, Masahiko Takai, Hideaki Kaneshige, Hideo Machimura, Akira Kanamori, Kazumi Nakajima, Ikuro Matsuba

**Affiliations:** a Diabetes Committee Study Group, Kanagawa Physicians Association, Kanagawa, Japan; b Nakajima Clinic, Kanagawa, Japan

**Keywords:** Type 2 diabetes, Dipeptidyl peptidase-4 inhibitor, Alogliptin, Hemoglobin A1c

## Abstract

**Background:**

Dipeptidyl peptidase-4 (DPP-4) inhibitors have been shown to reduce hemoglobin A1c (HbA1c) in patients with type 2 diabetes, but the reduction varies between patients and adequate glycemic control may not be achieved. We evaluated the efficacy and safety of the DPP-4 inhibitor alogliptin in the real clinical setting, and analyzed factors associated with the improvement of HbA1c by alogliptin treatment.

**Methods:**

A retrospective observational study was performed in patients with type 2 diabetes attending hospitals or clinics belonging to the Kanagawa Physicians Association who received treatment with alogliptin for 1 year or longer. Patients using insulin were excluded from the study. The efficacy endpoints were HbA1c (National Glycohemoglobin Standardization Program value), blood glucose (fasting/postprandial), body weight, blood pressure (systolic/diastolic), liver function (glutamate oxaloacetate transaminase, glutamate pyruvate transaminase, and γ-glutamyl transpeptidase), kidney function (serum creatinine and estimated glomerular filtration rate), serum lipids (total cholesterol, low-density lipoprotein cholesterol, high-density lipoprotein cholesterol, and triglycerides), and serum amylase. Adverse events were compiled to assess safety.

**Results:**

Of 330 patients whose case records were collected, 27 patients were excluded for protocol violations, leaving 303 patients to form the full analysis set. Compared with baseline, HbA1c showed a decrease by 0.54±1.22% (mean ± standard deviation) after 12 months of alogliptin treatment. Factor analysis demonstrated that the change of HbA1c after 12 months was significantly influenced by the baseline HbA1c level, duration of diabetes, concomitant use of sulfonylureas, and compliance with diet therapy. In addition, there was a significant reduction of total cholesterol, low-density lipoprotein cholesterol, and the estimated glomerular filtration rate after 12 months of alogliptin treatment, as well as a significant increase in serum creatinine. No significant changes of body weight, blood pressure, or liver function were observed. Symptoms of hypoglycemia occurred in two patients (0.6%).

**Conclusions:**

Alogliptin displayed a significant hypoglycemic effect and excellent safety in routine clinical use. Factors influencing the change of HbA1c with alogliptin therapy may include the HbA1c at the start of treatment, the duration of diabetes, use of sulfonylureas, and compliance with diet therapy.

## Introduction

Dipeptidyl peptidase-4 (DPP-4) inhibitors are a new class of oral antidiabetic agents that increase endogenous incretin levels and stimulate glucose-dependent insulin secretion by selectively inhibiting DPP-4, an enzyme that degrades circulating incretins (glucagon-like peptide-1 and glucose-dependent insulinotropic polypeptide) [1]. In 2009, sitagliptin was the first of these drugs to be approved in Japan and eight DPP-4 inhibitors are available as of 2015. Meta-analyses have shown that there is no significant difference of the hypoglycemic effect between DPP-4 inhibitors [2, 3]. These drugs have a good safety profile with a low risk of causing hypoglycemia or weight gain [4].

Alogliptin is a DPP-4 inhibitor that was marketed in Japan in 2010 [5]. A meta-analysis of the efficacy of alogliptin showed that hemoglobin A1c (HbA1c) was decreased by 0.81% (at a dose of 12.5 mg) and by 0.98% (at a dose of 25.0 mg) in patients treated with this drug compared with controls [6]. In addition, a large-scale comparative study (the EXAMINE study) found no difference in the risk of cardiovascular events between alogliptin and placebo group in patients with type 2 diabetes mellitus (T2DM) who had a history of acute coronary syndrome [7].

While DPP-4 inhibitors reduce HbA1c, the extent of the reduction varies between patients and some patients do not achieve adequate glycemic control. A meta-analysis of factors associated with HbA1c reduction indicated that baseline HbA1c and fasting blood glucose levels were useful predictors of the response [8]. Additionally, a meta-analysis of racial differences revealed that the reduction of HbA1c by DPP-4 inhibitors was greater in Asians than in non-Asians, and that body mass index (BMI) had a significant influence [9].

It has been reported that DPP-4 inhibitors can have lipid-lowering [10] and renoprotective [11] effects, in addition to their hypoglycemic effect. However, there were no significant difference in the changes of the lipid profile or estimated glomerular filtration rate (eGFR) between alogliptin and placebo in the EXAMINE study [7].

In order to evaluate the efficacy and safety of alogliptin in the real clinical setting, a retrospective observational study was conducted for 1 year from the start of alogliptin treatment in patients with T2DM who were attending clinics/hospitals belonging to the Kanagawa Physicians Association. The primary efficacy endpoint was the change of HbA1c after 12 months of treatment, and factor analysis was performed to identify patient characteristics associated with the improvement of HbA1c that could be used to predict efficacy.

## Patients and Methods

### Study design

A multicenter retrospective observational study was conducted at clinics and hospitals belonging to the Kanagawa Physicians Association. Data were collected from the medical records of the subjects and the follow-up period was 1 year. This study was approved by the Ethics Review Board of the Kanagawa Physicians Association.

### Patients

Patients were eligible for this study if they had T2DM, were aged 20 years old or older, regularly attended a clinic or hospital belonging to the Kanagawa Physicians Association, and received treatment with alogliptin for 1 year or longer. Alogliptin was started if glycemic control was inadequate for at least 1 month despite diet and exercise therapy or diet and exercise plus oral antidiabetic drugs.

The exclusion criteria were as follows: a history of hypersensitivity to any component of alogliptin; a history of severe ketoacidosis, diabetic coma or precoma within 6 months before the start of alogliptin therapy; severe infection; recent or planned surgery or severe trauma; concurrent use of insulin preparations or glinides; and patients who the attending doctor considered to be inappropriate for this study for other reasons.

### Items investigated

The baseline characteristics investigated for the subjects included the gender, age, height, duration of diabetes, family history, smoking history, alcohol history, and complications. Use of the following drugs was assessed before treatment with alogliptin, at the start of treatment, and 3 months, 6 months, 9 months, and 12 months after the start of treatment: alogliptin and other antidiabetic drugs, lipid-lowering drugs, and antihypertensive drugs. Efficacy endpoints were determined at each of the specified times, including HbA1c (National Glycohemoglobin Standardization Program value), blood glucose (fasting and postprandial), body weight (BW), blood pressure (BP; systolic/diastolic), liver function parameters (glutamate oxaloacetate transaminase, glutamate pyruvate transaminase, and γ-glutamyl transpeptidase), kidney function parameters (serum creatinine, eGFR), serum lipids (total cholesterol (TC), low-density lipoprotein (LDL) cholesterol, high-density lipoprotein cholesterol, triglycerides (TG)), and serum amylase. In addition, adverse events were evaluated at each of these times to assess safety.

### Statistical analysis

After patients who did not receive the study drug were excluded from those whose case records were collected, the remaining patients formed the safety analysis set. The full analysis set (FAS) was obtained by excluding patients who met any of the following criteria from the safety analysis set: 1) no HbA1c data at the start of alogliptin treatment, 2) no HbA1c data after the start of alogliptin treatment, or 3) administration of another DPP-4 inhibitor after the start of alogliptin treatment.

Appropriate descriptive statistics were calculated for the baseline characteristics of the FAS, including the gender, age, duration of diabetes, BMI, and BP.

Descriptive statistics were also calculated for the daily dose of alogliptin at each time of assessment, and for the use or non-use of other antidiabetic drugs, lipid-lowering drugs, and antihypertensive drugs at each assessment time.

Furthermore, descriptive statistics were calculated for all of the efficacy endpoints at each time of assessment. The following statistical analyses were performed. Linear mixed-effects models for repeated measures were employed to evaluate differences between each assessment time compared with the start of alogliptin treatment using the time of assessment as the fixed effect. Adjustment for multiplicity due to the number of assessment time points was done by the Dunnett-Hsu method. The percentage of patients who achieved specific HbA1c target values (< 6.0%, < 7.0%, or < 8.0%) was calculated at each time of assessment, and McNemar’s test was used to evaluate differences in the percentage at each time compared with the start of alogliptin treatment. Two multiple regression analysis models, which were a model using five baseline characteristics as explanatory variables (model 1) and a model using those variables plus concurrent treatment (in month 12) as the variables (model 2), were employed to analyze factors associated with the change of HbA1c (in month 12).

In the safety analysis set, the number and percentage of patients who developed adverse events (symptoms of hypoglycemia, constipation, and other events) and the number of episodes of each adverse event were tallied.

## Results

### Disposition of the subjects

**Figure 1:**
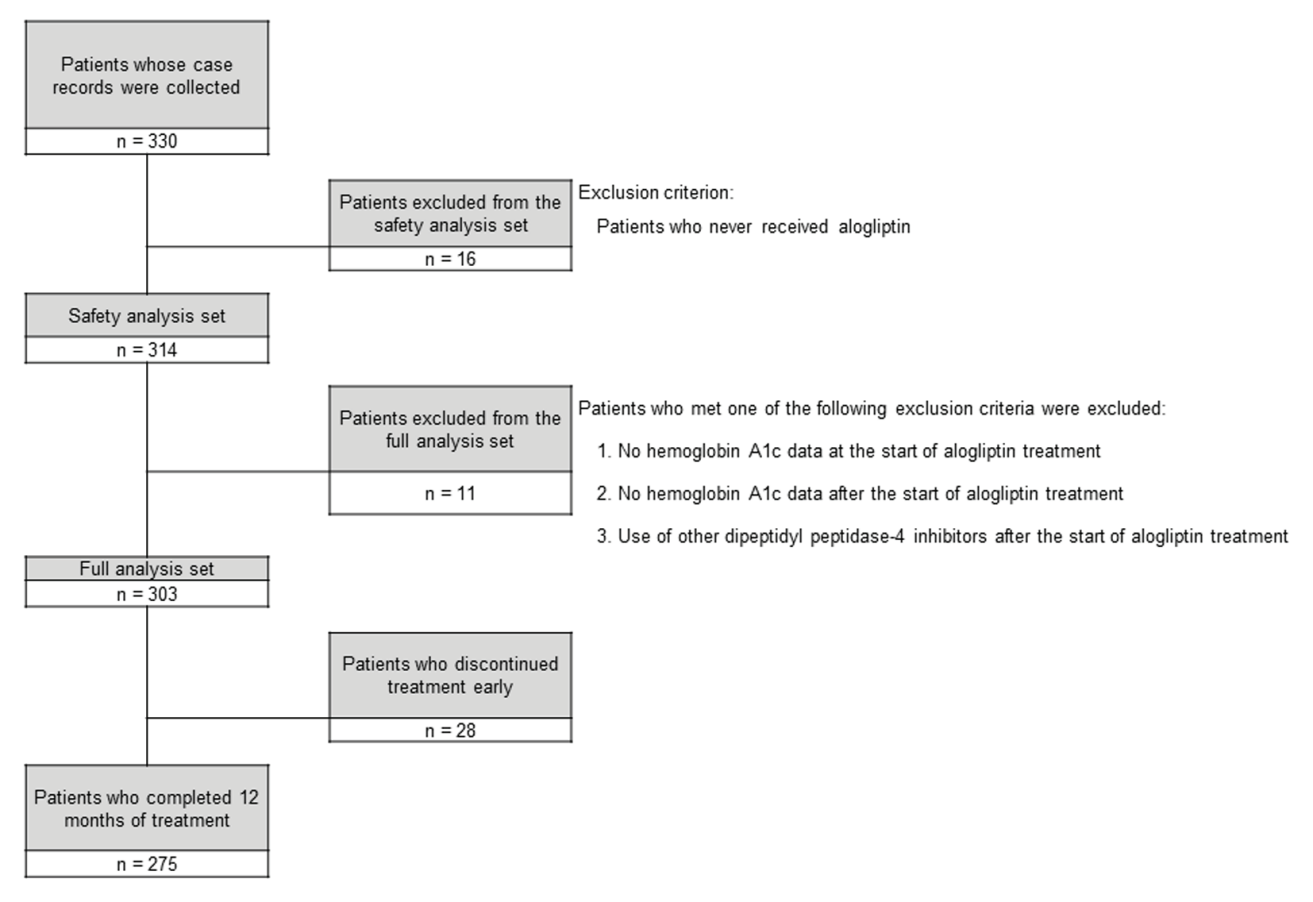
Disposition of the subjects.

The disposition of the subjects is shown in Figure 1. Of 330 patients whose case records were collected, 16 patients who did not receive alogliptin were excluded and the remaining 314 patients formed the safety analysis set. Then 11 patients who met any of the exclusion criteria for the FAS were excluded from those in the safety analysis set, leaving 303 patients in the FAS. Of these 303 patients, 28 patients discontinued alogliptin early and 275 patients completed 12 months of treatment.

### Baseline characteristics

Table 1 provides a summary of the baseline characteristics of the patients in the FAS (n = 303). Their mean age was 67.3 years and there was male predominance (56.1%). The mean duration of diabetes was 10.3 years and the diabetic complications included retinopathy (7.6%), neuropathy (8.6%), and nephropathy (11.2%). Regarding other complications, hypertension (59.4%) and dyslipidemia (55.8%) showed a high prevalence.

**Table 1:** Baseline Characteristics of the Full Analysis Set (N = 303)

Item	Male, 170 (56.1%), mean ± SD	Female, 127 (41.9%), median (range)	Unknown, 6 (2.0%), N
At the start of administration			
Age (years)	67.3 ± 11.9	69.0 (29 - 91)	290
Height (cm)	160.70 ± 9.93	161.00 (137.4 - 184.0)	235
Weight (kg)	64.814 ± 14.065	63.300 (37.10 - 130.00)	281
Body mass index (kg/m^2^)	24.98 ± 4.10	24.66 (16.3 - 41.5)	233
Systolic blood pressure (mm Hg)	135.2 ± 18.6	132.0 (98 - 213)	297
Diastolic blood pressure (mm Hg)	76.2 ± 11.5	76.0 (48 - 124)	297
Hemoglobin A1c (%)	7.37 ± 1.21	7.10 (4.8 - 11.9)	303
Blood glucose (fasting) (mg/dL)	152.4 ± 47.7	143.5 (77 - 339)	86
Blood glucose (postprandial) (mg/dL)	184.4 ± 66.4	176.0 (66 - 516)	105
Estimated duration of diabetes (years)	10.3 ± 7.6	9.0 (0 - 40)	176
	Yes	No	Unknown/no data
Family history	49 (16.2%)	94 (31.0%)	160 (52.8%)
Smoking history	58 (19.1%)	118 (38.9%)	127 (41.9%)
Alcohol history	86 (28.4%)	98 (32.3%)	119 (39.3%)
Complications			
Diabetic retinopathy	23 (7.6%)	225 (74.3%)	55 (18.2%)
Diabetic neuropathy	26 (8.6%)	222 (73.3%)	55 (18.2%)
Diabetic nephropathy	34 (11.2%)	219 (72.3%)	50 (16.5%)
Cerebrovascular accident (cerebral infarction/cerebral hemorrhage)	21 (6.9%)	224 (73.9%)	58 (19.1%)
Myocardial infarction	17 (5.6%)	228 (75.2%)	58 (19.1%)
Angina	33 (10.9%)	213 (70.3%)	57 (18.8%)
Arteriosclerosis obliterans (lower limbs)	10 (3.3%)	222 (73.3%)	71 (23.4%)
Hypertension	180 (59.4%)	83 (27.4%)	40 (13.2%)
Dyslipidemia	169 (55.8%)	90 (29.7%)	44 (14.5%)
Fatty liver	49 (16.2%)	178 (58.7%)	76 (25.1%)
Others	76 (25.1%)	153 (50.5%)	74 (24.4%)

### Medications

The medications used by the patients in the FAS (n = 303) are shown in Table 2. Before the start of treatment with alogliptin, 74.3% of the patients were using one or more antidiabetic drugs. Drugs taken by 10% or more of the patients included glimepiride (31.0%), other DPP-4 inhibitors (26.4%), α-glucosidase inhibitors (24.8%), metformin (24.1%), and pioglitazone (19.5%). At the start of alogliptin treatment, the patients were not using other DPP-4 inhibitors and the percentage of patients using α-glucosidase inhibitors or glinides showed a marked decrease from 24.8% to 15.8% and from 5.9% to 0.3%, respectively. On the other hand, the use of metformin increased after the start of alogliptin treatment and 34.9% of the patients were taking it after 12 months.

**Table 2:** Medications of the Full Analysis Set

	Assessment times (baseline = start of alogliptin treatment)					
	Before administration	Baseline	Month 3	Month 6	Month 9	Month 12
Patients receiving the study drug in the full analysis set	303 (100.0%)	303 (100.0%)	303 (100.0%)	290 (100.0%)	278 (100.0%)	275 (100.0%)
Antidiabetic drugs						
Total	225 (74.3%)	303 (100.0%)	303 (100.0%)	290 (100.0%)	278 (100.0%)	275 (100.0%)
Alogliptin	-	303 (100.0%)	303 (100.0%)	290 (100.0%)	278 (100.0%)	275 (100.0%)
DPP-4 inhibitors	80 (26.4%)	0 (0.0%)	0 (0.0%)	0 (0.0%)	0 (0.0%)	0 (0.0%)
Glimepiride	94 (31.0%)	90 (29.7%)	95 (31.4%)	87 (30.0%)	81 (29.1%)	83 (30.2%)
Glibenclamide	10 (3.3%)	7 (2.3%)	7 (2.3%)	7 (2.4%)	7 (2.5%)	7 (2.5%)
Gliclazide	15 (5.0%)	13 (4.3%)	14 (4.6%)	16 (5.5%)	16 (5.8%)	17 (6.2%)
Metformin	73 (24.1%)	82 (27.1%)	91 (30.0%)	91 (31.4%)	93 (33.5%)	96 (34.9%)
Pioglitazone	59 (19.5%)	52 (17.2%)	60 (19.8%)	56 (19.3%)	50 (18.0%)	46 (16.7%)
α-Glucosidase inhibitors	75 (24.8%)	48 (15.8%)	48 (15.8%)	45 (15.5%)	40 (14.4%)	38 (13.8%)
Glinides	18 (5.9%)	1 (0.3%)	1 (0.3%)	1 (0.3%)	1 (0.4%)	1 (0.4%)
Insulin	1 (0.3%)	0 (0.0%)	0 (0.0%)	0 (0.0%)	1 (0.4%)	1 (0.4%)
Lipid-lowering agents						
Total	133 (43.9%)	140 (46.2%)	145 (47.9%)	146 (50.3%)	144 (51.8%)	143 (52.0%)
Statins	116 (38.3%)	123 (40.6%)	129 (42.6%)	131 (45.2%)	130 (46.8%)	129 (46.9%)
Others	29 (9.6%)	29 (9.6%)	31 (10.2%)	31(10.7%)	31(11.2%)	32 (11.6%)
Antihypertensive agents						
Total	168 (55.4%)	174 (57.4%)	177 (58.4%)	176 (60.7%)	171 (61.5%)	174 (63.3%)
ARB	122 (40.3%)	125 (41.3%)	127 (41.9%)	128 (44.1%)	124 (44.6%)	127 (46.2%)
Ca antagonists	113 (37.3%)	116 (38.3%)	119 (39.3%)	117 (40.3%)	116 (41.7%)	118 (42.9%)
Diuretics	22 (7.3%)	20 (6.6%)	23(7.6%)	23(7.9%)	21 (7.6%)	22 (8.0%)
ACE inhibitors	14 (4.6%)	15 (5.0%)	14 (4.6%)	13 (4.5%)	13 (4.7%)	13 (4.7%)
Renin inhibitors	4 (1.3%)	3 (1.0%)	0 (0.0%)	0 (0.0%)	0 (0.0%)	0 (0.0%)
α-blockers	8 (2.6%)	8 (2.6%)	10 (3.3%)	10 (3.4%)	10 (3.6%)	11 (4.0%)
β-blocker	6 (2.0%)	6 (2.0%)	7 (2.3%)	8 (2.8%)	8 (2.9%)	8 (2.9%)
αβ-blockers	11 (3.6%)	13 (4.3%)	13 (4.3%)	13 (4.5%)	12 (4.3%)	11 (4.0%)
Aldosterone blockers	3 (1.0%)	3 (1.0%)	4 (1.3%)	3(1.0%)	3 (1.1%)	3 (1.1%)
Others	0 (0.0%)	0 (0.0%)	0 (0.0%)	0 (0.0%)	0 (0.0%)	0 (0.0%)

ACE: angiotensin-converting enzyme; ARB: angiotensin receptor blocker; DPP-4: dipeptidyl peptidase-4.

Before the start of alogliptin treatment, 43.9% of the patients were taking lipid-lowering drugs and 55.4% were taking antihypertensive agents, with these percentages increasing to 52.0% and 63.3%, respectively, at 12 months after the start of alogliptin treatment.

**Figure 2:**
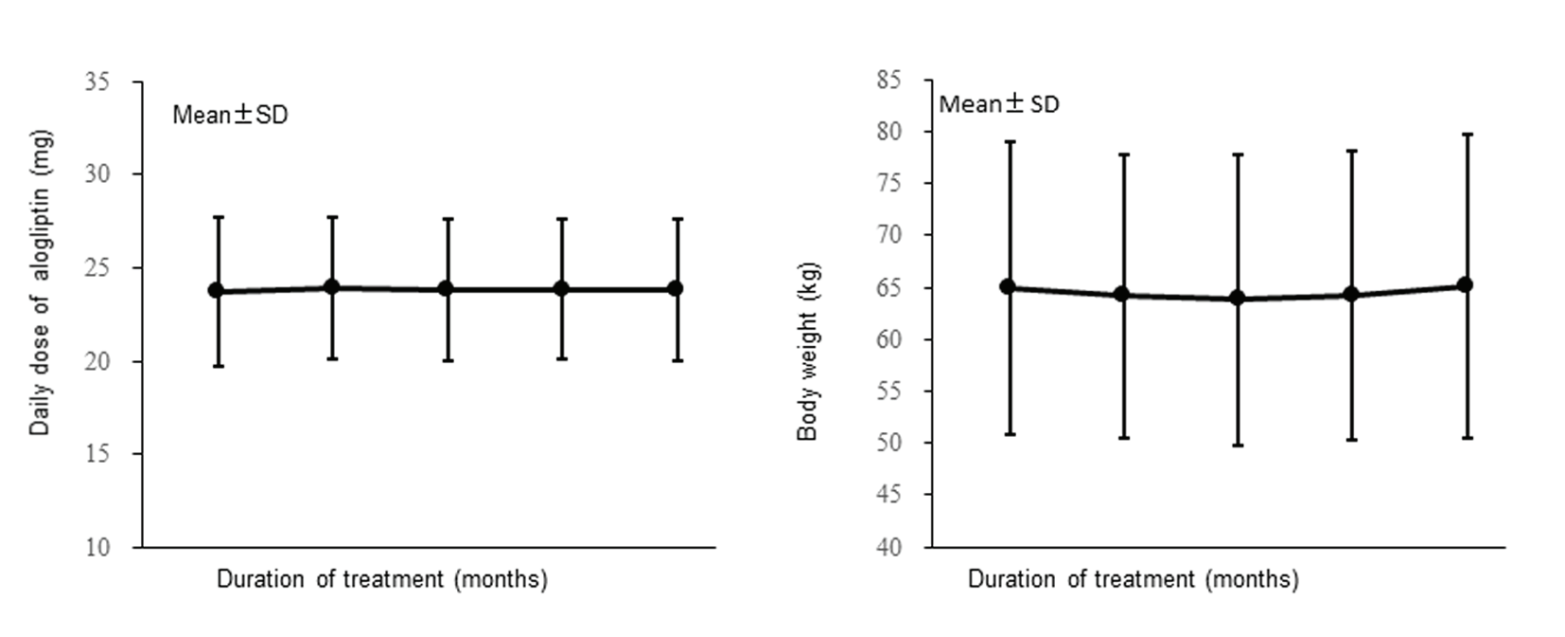
Changes of the daily dose of alogliptin and the body weight (full analysis set).

The mean daily dose of alogliptin is displayed in Figure 2 (left). It was 23.7 mg at the start of treatment and did not change significantly, being 23.8 mg after 12 months of treatment.

### Changes of HbA1c

**Figure 3:**
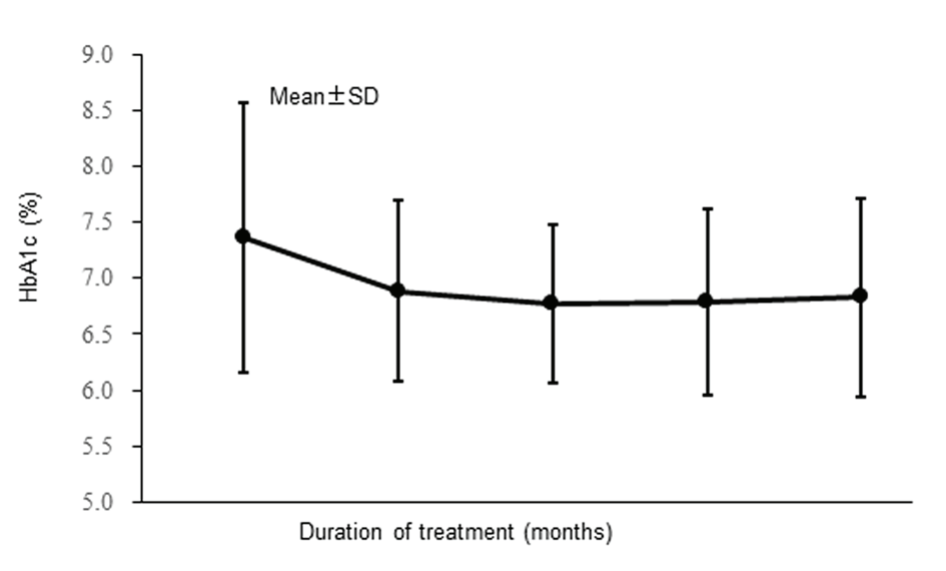
Changes of hemoglobin A1c (full analysis set).

The changes of HbA1c are shown in Figure 3. Mean HbA1c decreased from 7.37% at the start of alogliptin treatment to 6.78% after 6 months of treatment and did not change much after that, being 6.83% at 12 months. After12 months of alogliptin treatment, HbA1c (mean ± standard deviation) was reduced by 0.54±1.22% (n = 259).

The percentage of patients achieving the target HbA1c level was significantly larger at every time of assessment from 3 months after the start of alogliptin treatment compared with before alogliptin treatment (HbA1c was < 7.0% in 42.2% of patients before alogliptin treatment vs. 63.3% after 12 months; P < 0.001 by McNemar’s test).

The results of multiple regression analysis using the change of HbA1c after 12 months as a response variable are displayed in Table 3. Model 1 (using baseline characteristics as the explanatory variables) was employed for analysis of 110 patients from the FAS with complete data on the age, disease duration, and BMI. This analysis showed that the disease duration and the baseline HbA1c level had a significant influence on the change of HbA1c after 12 months. The reduction of HbA1c was larger as the disease duration became shorter and as baseline HbA1c increased.

**Table 3:** Multiple Regression Analysis of Changes in Hemoglobin A1c After 12 Months (Full Analysis Set)

Explanatory variables	Model 1: baseline characteristics (N = 110)					Model 2: baseline characteristics + concurrent treatment (N = 76)				
	Estimate	Standard error	95% confidence interval		P value	Estimate	Standard error	95% confidence interval		P value
			Lower limit	Upper limit				Lower limit	Upper limit	
Baseline characteristics										
Gender (female = 0; male = 1)	0.0213	0.1683	-0.3125	0.3551	0.8996	-0.0983	0.1833	-0.4645	0.2679	0.5935
Age (years)	-0.0107	0.0083	-0.0271	0.0056	0.1963	-0.0168	0.0092	-0.0352	0.0015	0.0718
Duration of diabetes (years)	0.0366	0.0113	0.0143	0.0589	0.0015*	0.0156	0.0134	-0.0112	0.0425	0.2490
Hemoglobin A1c (%)	-0.8660	0.0729	-1.0106	-0.7214	< 0.0001*	-0.9264	0.0749	-1.0761	-0.7767	< 0.0001*
Body mass index (kg/m^2^)	0.0001	0.0243	-0.0481	0.0482	0.9983	-0.0207	0.0244	-0.0695	0.0281	0.4007
Use of concomitant drugs in month 12: no = 0; yes = 1										
Sulfonylureas						0.4211	0.1897	0.0420	0.8001	0.0300*
Biguanides						0.2787	0.1911	-0.1030	0.6605	0.1496
Thiazolidinediones						-0.1891	0.2282	-0.6450	0.2667	0.4102
α-Glucosidase inhibitors						-0.1456	0.2218	-0.5887	0.2976	0.5140
Compliance with concurrent treatment in month 12: poor = 0; fair or good = 1										
Diet therapy						-1.2210	0.3144	-1.8490	-0.5929	0.0002*
Exercise therapy						-0.4497	0.2735	-0.9961	0.0968	0.1051

*P < 0.050.

Then analysis was performed using model 2 (adding concurrent treatment to the explanatory variables of model 1) in the patients from model 1 with information on concurrent treatment at 12 months. This analysis showed that use or non-use of sulfonylureas at 12 months and compliance with diet therapy significantly influenced the change of HbA1c, in addition to the influence of baseline HbA1c. When sulfonylureas were not being used after 12 months and compliance with diet was better, the reduction of HbA1c at 12 months was larger. While the duration of diabetes had a significant influence on HbA1c in model 1, there was no significant effect in model 2.

### Other efficacy endpoints

Changes of BW over time in the FAS are shown in Figure 2 (right). Mean BW did not change significantly, being 64.95 kg before the start of alogliptin treatment and 65.10 kg after 12 months of treatment.

The results for the other efficacy endpoints are listed in Tables 4-6. After 12 months of alogliptin treatment, serum creatinine showed a significant increase, while fasting blood glucose, eGFR, TC, and LDL cholesterol were all significantly decreased.

**Table 4:** Efficacy Endpoints in the Full Analysis Set: Blood Glucose, Body Mass Index, and Blood Pressure

Item	Time after starting alogliptin (months)	Measured values						Changes of measured values				
		N	Mean	SD	Median	Maximum	Minimum	Point estimate	SE	95% confidence interval		P value‡
										Lower limit	Upper limit	
Blood glucose (fasting) (mg/dL)	0	98	150.2	46.9	339	140.0	77	-	-	-	-	-
	3	79	137.8	38.4	294	131.0	80	-11.07	5.08	-23.53	1.39	0.0954
	6	71	131.4	27.9	204	130.0	72	-16.39	3.64	-25.34	-7.45	< 0.0001*
	9	73	135.2	35.7	290	127.0	90	-15.26	4.77	-26.96	-3.56	0.0062*
	12	68	133.6	41.9	315	123.5	84	-16.79	5.46	-30.19	-3.38	0.0092*
Blood glucose (postprandial) (mg/dL)	0	118	185.7	68.1	516	174.5	66	-	-	-	-	-
	3	107	158.6	45.7	327	154.0	83	-23.27	5.50	-36.73	-9.81	0.0002*
	6	108	161.7	59.5	364	147.5	71	-19.05	5.72	-33.05	-5.06	0.0040*
	9	93	163.9	54.5	362	152.0	59	-14.31	6.66	-30.62	1.99	0.1009
	12	114	170.6	60.9	413	155.0	76	-10.76	6.52	-26.70	5.19	0.2705
Body mass index (kg/m^2^)	0	230	25.04	4.08	41.5	24.67	16.3	-	-	-	-	-
	3	187	24.76	4.02	39.8	24.40	15.6	-0.07	0.05	-0.20	0.07	0.5238
	6	176	24.81	4.03	38.1	24.49	15.2	-0.02	0.09	-0.25	0.22	0.9992
	9	155	24.71	3.80	39.2	24.47	15.0	0.04	0.08	-0.16	0.24	0.9572
	12	175	25.00	4.19	43.6	24.73	15.0	0.00	0.09	-0.21	0.21	1.0000
Systolic blood pressure (mm Hg)	0	297	135.2	18.6	213	132.0	98	-	-	-	-	-
	3	286	133.0	15.7	183	132.0	94	-2.15	0.94	-4.43	0.13	0.0704
	6	265	133.7	16.2	197	132.0	98	-1.64	0.98	-4.02	0.74	0.2527
	9	248	132.6	14.8	193	132.0	97	-2.60	1.09	-5.23	0.04	0.0546
	12	261	133.0	16.1	211	132.0	90	-2.19	1.10	-4.85	0.47	0.1349
Diastolic blood pressure (mm Hg)	0	297	76.2	11.5	124	76.0	48	-	-	-	-	-
	3	286	75.4	10.8	110	75.0	45	-0.64	0.63	-2.18	0.90	0.6896
	6	264	74.9	10.4	112	75.0	43	-1.16	0.59	-2.60	0.27	0.1480
	9	247	74.7	10.7	112	74.0	50	-1.47	0.68	-3.14	0.19	0.0994
	12	261	75.4	10.4	102	75.0	50	-0.88	0.59	-2.31	0.56	0.3626

‡Linear mixed-effects models (covariance structure = unstructured) were used and multiplicity was adjusted by the Dunnett-Hsu method. *P < 0.050. SD: standard deviation; SE: standard error.

**Table 5:** Efficacy Endpoints in the Full Analysis Set: Liver Function and Kidney Function

Item	Time after starting alogliptin (months)	Measured values						Changes of measured values				
		N	Mean	SD	Median	Maximum	Minimum	Point estimate	SE	95% confidence interval		P value‡
										Lower limit	Upper limit	
GOT (IU/L)	0	217	27.8	16.4	136	22.0	11	-	-	-	-	-
	3	176	27.2	14.8	112	22.0	12	-1.32	0.86	-3.41	0.77	0.3332
	6	171	26.1	13.1	97	21.0	11	-1.21	1.01	-3.68	1.26	0.5509
	9	132	27.2	16.9	143	23.0	11	-0.06	1.03	-2.58	2.47	1.0000
	12	164	27.5	16.3	122	23.0	12	0.16	1.21	-2.79	3.12	0.9997
GPT (IU/L)	0	239	29.8	24.5	179	21.0	6	-	-	-	-	-
	3	205	27.8	20.7	138	20.0	6	-2.48	0.99	-4.91	-0.06	0.0432*
	6	190	27.5	24.2	237	20.0	5	-2.27	1.48	-5.90	1.36	0.3482
	9	157	27.4	17.9	109	21.0	8	-2.04	1.13	-4.82	0.74	0.2160
	12	185	28.5	23.6	188	21.0	8	-1.77	1.37	-5.14	1.60	0.5063
γ-GTP (IU/L)	0	218	47.9	59.6	467	30.0	9	-	-	-	-	-
	3	177	48.2	63.1	407	29.0	8	-1.83	1.40	-5.28	1.62	0.5098
	6	166	48.7	55.8	365	30.0	8	-2.70	2.45	-8.75	3.34	0.6536
	9	136	56.5	88.5	754	29.0	9	1.58	3.88	-8.00	11.17	0.9842
	12	164	47.1	60.8	497	30.0	7	-1.55	3.83	-11.02	7.92	0.9846
Serum creatinine (mg/dL)	0	227	0.719	0.208	1.970	0.69	0.33	-	-	-	-	-
	3	193	0.753	0.283	3.23	0.730	0.31	0.023	0.007	0.006	0.041	0.0052*
	6	184	0.747	0.211	1.83	0.720	0.40	0.025	0.007	0.009	0.042	0.0008*
	9	161	0.738	0.228	2.10	0.720	0.33	0.025	0.007	0.009	0.042	0.0005*
	12	186	0.759	0.207	1.99	0.730	0.34	0.031	0.006	0.015	0.047	< 0.0001*
eGFR (mL/min/1.73 m^2^)	0	224	79.94	20.22	150.3	78.67	23.5	-	-	-	-	-
	3	192	77.79	22.5	220.1	76.09	15.6	-2.16	0.87	-4.28	-0.03	0.0451*
	6	181	76.48	18.66	129.4	74.24	29.2	-3.06	0.76	-4.92	-1.20	0.0003*
	9	160	78.96	19.89	159.7	78.60	25.1	-2.16	0.80	-4.12	-0.19	0.0263*
	12	183	75.78	18.95	155.5	74.16	26.7	-3.50	0.77	-5.41	-1.60	< 0.0001*

‡Linear mixed-effects models (covariance structure = unstructured) were used and multiplicity was adjusted by the Dunnett-Hsu method. *P < 0.050. eGFR: estimated glomerular filtration rate; γ-GTP: γ-glutamyl transpeptidase; GOT: glutamate oxaloacetate transaminase; GTP: glutamate pyruvate transaminase; SD: standard deviation; SE: standard error.

**Table 6:** Efficacy Endpoints in the Full Analysis Set: Lipids and Serum Amylase

Item	Time after starting alogliptin (months)	Measured values						Changes of measured values				
		N	Mean	SD	Median	Maximum	Minimum	Point estimate	SE	95% confidence interval		P value‡
										Lower limit	Upper limit	
Total cholesterol (mg/dL)	0	163	193.2	33.6	301	191.0	127	-	-	-	-	-
	3	143	188.6	31.7	326	186.0	113	-3.90	2.28	-9.49	1.69	0.2498
	6	127	186.8	31.2	283	186.0	114	-5.98	2.81	-12.88	0.92	0.1082
	9	108	182.8	30.5	252	182.5	105	-10.86	2.58	-17.20	-4.52	0.0002*
	12	119	187.1	31.9	289	186.0	104	-6.84	2.48	-12.93	-0.74	0.0227*
LDL cholesterol (mg/dL)	0	211	112.91	28.85	208.0	113.00	50	-	-	-	-	-
	3	174	109.13	26.88	198.0	108.00	47	-2.93	1.77	-7.26	1.40	0.2716
	6	165	107.62	27.95	198.0	110.00	56	-5.56	1.91	-10.21	-0.91	0.0136*
	9	135	104.84	24.95	163.0	104.00	48	-8.76	2.03	-13.70	-3.82	< 0.0001*
	12	159	107.04	24.97	186.0	107.00	57	-7.22	1.95	-11.99	-2.46	0.0010*
HDL cholesterol (mg/dL)	0	234	54.60	13.15	96.0	53.00	29	-	-	-	-	-
	3	199	54.34	13.52	100.0	52.00	27	0.20	0.49	-0.99	1.40	0.9826
	6	183	54.73	13.26	96.0	53.00	26	0.45	0.56	-0.93	1.84	0.8432
	9	159	54.31	14.26	98.0	52.00	26	0.06	0.61	-1.43	1.56	0.9999
	12	182	54.90	13.16	96.0	53.00	31	0.46	0.57	-0.94	1.87	0.8413
Triglycerides (mg/dL)	0	244	165.3	144.1	1520	136.5	34	-	-	-	-	-
	3	211	157.4	172.1	2190	124.0	32	-4.61	10.07	-29.23	20.00	0.9693
	6	195	152.2	109.4	951	127.0	28	-15.25	7.45	-33.46	2.97	0.1288
	9	171	147.0	87.9	619	124.0	27	-20.17	7.01	-37.31	-3.03	0.0151*
	12	194	144.2	79.3	528	125.0	35	-16.50	7.20	-34.10	1.09	0.0730
Serum amylase (IU/L)	0	58	66.5	24.9	121	60.0	31	-	-	-	-	-
	3	47	72.7	23.7	119	70.0	33	-	-	-	-	-
	6	36	74.5	23.8	121	72.5	24	-	-	-	-	-
	9	11	72.6	16.0	106	72.0	51	-	-	-	-	-
	12	22	71.9	27.2	131	70.0	33	-	-	-	-	-

‡Linear mixed-effects models (covariance structure = unstructured) were used and multiplicity was adjusted by the Dunnett-Hsu method. *P < 0.050. HDL: high-density lipoprotein; LDL: low-density lipoprotein; SD: standard deviation; SE: standard error.

### Safety

Twelve adverse events were reported in eight out of 314 patients (2.5%) in the safety analysis set. These adverse events included constipation (six events in three patients), hypoglycemia (two events in two patients), and fracture, neuropathy, hypertension, and lipid abnormality (each event occurred in one patient).

## Discussion

The present study investigated the efficacy and safety of alogliptin therapy in patients with T2DM who were attending hospitals or clinics belonging to the Kanagawa Physicians Association, employing efficacy endpoints such as the profile of HbA1c over time or the numerical change of HbA1c.

Of the 330 patients whose case records were collected, 27 patients were excluded from the FAS. Thus, a high proportion of all subjects were included in the FAS (91.8%; 303/330 patients).

Comparison of baseline characteristics between this study and a study of sitagliptin conducted in 1,332 patients [11] revealed that the sex ratio and BMI were similar (56.1% men in this study vs. 56.4% and mean BMI of 24.98 vs. 24.6), but the mean age (67.3 vs. 62.9 years old), mean systolic BP (135.2 mm Hg vs. 128.5 mm Hg), proportion of patients with hypertension (59.4% vs. 32%), and proportion of patients with dyslipidemia (55.8% vs. 36%) were higher in the present study. On the other hand, the mean duration of diabetes (10.3 vs. 12.0 years), mean HbA1c (7.37% vs. 8.0%), and proportion of patients with complications of diabetes (retinopathy, 7.6% vs. 32%; neuropathy, 8.6% vs. 26%; nephropathy, 11.2% vs. 28%) were lower in this study. Thus, compared with the patient population of the sitagliptin study, the age, BP, and lipid levels were higher and glycemic control was better at the start of alogliptin treatment in this study, while fewer patients had diabetic complications.

Before the start of alogliptin treatment, 74.3% of the patients were using antidiabetic drugs, including glimepiride (31.0%), other DPP-4 inhibitors (26.4%), α-glucosidase inhibitors (α-GIs) (24.8%), metformin (24.1%), and pioglitazone (19.5%). At the start of alogliptin treatment, 39.3% of the patients were not taking concomitant drugs, 34.0% were taking one drug, 17.5% were using two drugs, and 9.2% were on three drugs. The concomitant drugs included glimepiride (29.7%), metformin (27.1%), pioglitazone (17.2%), and α-GIs (15.8%). After 12 months of alogliptin treatment, 34.0% of the patients were not taking concomitant drugs, 34.5% were taking one drug, 24.0% were using two drugs, 6.5% were using three drugs, and 0.7% were on four drugs. Concomitant drugs included metformin (34.9%), glimepiride (30.2%), pioglitazone (16.72%), and α-GIs (13.8%).

HbA1c decreased significantly over time after the start of alogliptin treatment, and the mean reduction of HbA1c at 12 months was 0.54%. In a study of 1,057 patients, the mean reduction of HbA1c after 12 months of treatment with sitagliptin was reported to be 0.7% [12, 13]. The reduction of HbA1c was smaller in the present study, probably because baseline HbA1c levels were different (mean baseline HbA1c was 7.37% in this study vs. 8.0% in the sitagliptin study).

Multivariate analysis was performed using baseline characteristics as the explanatory variables (model 1) to identify factors associated with the change of HbA1c after 12 months, revealing that the baseline HbA1c and duration of diabetes had a significant independent influence on the change of HbA1c. Analysis was also performed after adding concurrent treatment to the explanatory variables (model 2), and identified concomitant use of sulfonamides and compliance with diet therapy as significant factors. In a previous study of 93 patients treated with sitagliptin, factor analysis revealed that noncompliance with diet therapy or exercise therapy were independent factors influencing the increase in HbA1c (by 0.3% or more) after 1.5 years [14].

In the present study, there were no significant changes of BW, BMI, BP (systolic/diastolic), and liver function (glutamate oxaloacetate transaminase, glutamate pyruvate transaminase, and γ-glutamyl transpeptidase) during alogliptin treatment. On the other hand, significant changes of lipids (TC and LDL cholesterol), kidney function (serum creatinine and eGFR), and the fasting blood glucose level were noted after 12 months of alogliptin treatment. In a study of 940 patients, TC showed a significant decrease and serum creatinine was increased significantly by sitagliptin treatment [15]. In addition, evaluation of serum creatinine up to 2 years (n = 826) showed that the serum creatinine level was increased significantly at 1 month after the start of sitagliptin treatment, but there were no significant changes subsequently [16].

There were 314 patients in the safety analysis set of the present study. The incidence rates of hypoglycemic symptoms and constipation were 0.6% (n = 2) and 1.0% (n = 3), respectively, so the data demonstrated that alogliptin was a very safe drug.

With regard to limitations, this was an observational study without a control group. When interpreting the results of the study, it should be noted that changes observed after the start of alogliptin treatment were not necessarily due to the effects of alogliptin.

### Conclusion

HbA1c and fasting blood glucose levels were significantly reduced by treatment with alogliptin, demonstrating that this drug had a clinically useful hypoglycemic effect. Analysis of the changes of HbA1c suggested that the baseline HbA1c level, duration of diabetes, concomitant use of sulfonylureas, and compliance with diet therapy influenced the reduction of HbA1c by alogliptin. In addition, there was a significant reduction of TC and LDL cholesterol levels, suggesting that alogliptin had a lipid-lowering effect. Furthermore, a significant increase in serum creatinine and significant reduction of eGFR suggested that alogliptin influences kidney function. Alogliptin treatment was safe, with little change of BW and a low incidence of hypoglycemia (0.6%).
